# The perspectives of oncology healthcare providers on the role of palliative care in a comprehensive cancer center

**DOI:** 10.1186/s12904-022-01039-7

**Published:** 2022-08-24

**Authors:** Trenley M. Anderson, Megan M. Farrell, Gabriel Moss, Mona Gupta, Stefanie Mooney, Katherine Daunov, Megan Savernick, Jan Frandsen, Kolby Verrona, Aryn Pecoraro, Cassandra Mance, Jorge Garcia, Richard T. Lee

**Affiliations:** 1 Case Western Reserve University School of Medicine, Cleveland, OH USA; 2 University Hospitals, Cleveland, OH USA; 3 City of Hope Comprehensive Cancer Center, Duarte, CA USA

**Keywords:** Palliative care, Oncology, Survey, Barriers, Inpatient, Outpatient

## Abstract

**Background:**

Palliative care (PC) is an essential part of oncologic care, but its optimal role within a cancer center remains unclear. This study examines oncology healthcare providers’ perspectives about the role of PC at a comprehensive cancer center (CCC).

**Methods:**

Physicians, nurses, and other oncology healthcare providers at a CCC were surveyed for their opinions about the role of inpatient and outpatient PC, preferences for PC services, and barriers to referral. Chi-squared tests and multiple regression analyses were performed to explore associations.

**Results:**

We received 137/221 completed questionnaires (61% response rate). Respondents were generally female (78%), had ≤ 10 years of service (69%), and included physicians (32%), nurses (32%), and advanced practice providers (17%). Most respondents (82%) agreed that more patients could benefit from PC. They also agreed that PC is beneficial for both outpatient and inpatient management of complex pain (96 and 88%), complex symptoms (84 and 74%), and advanced cancer patients (80 and 64%). Transition to hospice (64 vs. 42%, *p* = 0.007) and goals of care (62 vs. 49%, *p* = 0.011) provided by PC services were more valued by respondents for the inpatient than for the outpatient setting. Barriers to utilizing PC included lack of availability, unsure of when to refer, and poor communication. The majority of respondents (83%) preferred a cancer focused PC team to provide high-quality care.

**Conclusions:**

Overall, the majority of oncology health care providers believe that more patients could benefit from PC, but opinions vary regarding the roles of inpatient and outpatient PC. Barriers and areas for improvement include availability, referral process, and improved communication.

**Supplementary Information:**

The online version contains supplementary material available at 10.1186/s12904-022-01039-7.

## Background

The World Health Organization defines palliative care (PC) as an approach that improves the quality of life of patients and their families by preventing and relieving suffering through the assessment and treatment of pain and a variety of other symptoms, whether physical, psychological, social, or spiritual [1]. PC involves a variety of services delivered by a wide range of professionals including physicians, nurses, chaplains, social workers, and other supportive care providers. Multiple randomized controlled trials have shown that early incorporation of PC interventions for advanced cancer patients is beneficial for both quality of life and for survival [2–4]. In response to these findings, the American Society of Clinical Oncology (ASCO) has published recommendations for integration of PC early in the course of disease for cancer patients and is recognized as an essential service within cancer centers, which have been supported by other national organizations [5–7].

Implementation of these recommendations, however, remains challenging due to a variety of barriers. The most-reported barriers are system-related and include limited availability of PC services, poor communication between teams, lack of interdisciplinary communication, and low insurance reimbursement [8, 9]. Given these challenges, the best models for PC integration at comprehensive cancer centers (CCC) are still being evaluated. Conceptual models of integration have been discussed, but additional research is still needed to determine best practices [10].

We were interested in discovering ways to grow and improve the PC program at our institution, a National Cancer Institute designated CCC. At our institution, the inpatient and outpatient PC services were provided separately at the time of this study, and aimed to evaluate if PC roles differ between these settings. To date, few surveys have focused on the role of PC in inpatient versus outpatient settings among a variety of healthcare professionals at a CCC. Thus, as part of a quality improvement initiative, we surveyed the perspectives of a variety of oncology providers to better define the perceived role of PC and to assess barriers at our institution with an ultimate goal of how do we improve the PC service within the cancer center.

## Methods

The survey was created by RL for the purposes of informing the design of the PC program at our CCC. Initial drafts were reviewed by members of the PC program. Respondents were surveyed about the role of PC for cancer patients in both inpatient and outpatient settings and preferences regarding PC team structure and specialization. The palliative care service at the time of this survey included both physicians and nurse practitioners. Two separate teams provided care in the outpatient clinic versus the inpatient hospital. Other supportive care services (social work, chaplaincy, psychiatry) were being provided within the CCC but were not explicitly included. The survey asked whether patients with the following characteristics would benefit most from outpatient and inpatient PC consultation (check all that apply): end of life, active cancer treatment, advanced cancer, complex pain, complex symptoms, or unclear goals of care. Respondents were asked about the frequency of outpatient/inpatient referral to PC and whether any of the following were barriers to referral: lack of PC availability, poor communication, poor alignment with oncology team’s care plan, lack of understanding of cancer care, poor coordination of care, poor ability to address advanced care planning or goals of care, poor ability to manage complex symptoms or complex pain, poor continuity of care between inpatient and outpatient, poor ability to provide emotional or spiritual support, lack of trust with PC providers, uncertainty about when to refer, uncertainty about how to refer, and lack of time to make a referral.

Likert scales were utilized when applicable when asking about, outpatient and inpatient PC team coordination of care, quality of communication, and quality of care (excellent, very good, good, fair, poor), and overall satisfaction with outpatient and inpatient PC teams (very satisfied, satisfied, moderately satisfied, somewhat satisfied, not satisfied). A Likert scale (strongly disagree, disagree, neutral, agree, strongly agree) was also used to assess (1) whether respondents felt that more patients could benefit from PC, (2) whether having the same PC clinicians seeing patients in both outpatient and inpatient settings is important for continuity of care, (3) and for quality of care, (4) whether having a team of PC clinicians that focuses on cancer is important for high-quality care, and (5) whether respondents would prefer a team of PC clinicians that focuses on cancer (as compared to a PC team that sees all types of medical conditions). Subjects were asked to describe frequency of referral to outpatient/inpatient PC. The survey also asked respondents to report their demographics, professional role, practice location, years of service at our CCC, and types of cancer they treat. Most of the questions allowed for an other option with free text. These responses were limited and thus not included in this publication.

We administered the survey from December 2018 to January 2019 as part of a quality improvement project. The survey was emailed to physicians, advanced practice providers (APP), nurses, and social workers at a National Cancer Institute designated CCC in Northeast Ohio. All responses were collected anonymously with REDCap®. Respondents were provided $5–10 compensation. All experimental protocols were approved by the IRB of University Hospitals.

All data were pre-coded by REDCap® and checked for errors by the research team. Missing and ambiguous responses were excluded from the analysis. Years of service, location, and cancer specialty groups were combined into more condensed groups for analyses. Descriptive statistics (i.e., frequency, mean) were used to summarize oncology health care professional characteristics and outcome variables. Chi-square tests were performed to explore categorical comparisons.

Along with the survey’s summary statistics, a multivariable regression analysis was conducted on the referral frequency (< 1x/month vs. ≥ 1x/month), agree that more patients would benefit from PC (agree vs. no agreement) service ratings, prefer a cancer focused PC team (agree vs. no agreement), and an automatic assessment would be helpful for PC referral (agree vs. no agreement) in a logistics regression model. The model included gender (male versus female), type of healthcare provider (physician vs. non-physician), specialty (general oncology vs. specialized medical oncology vs. hematology), location (main campus vs. community), and years of service (< 10 vs 11–25 vs 26–41 vs > 41 years). All statistical analyses were performed using Stata/SE 14.2 (College Station, TX).

## Results

### Demographics

Of the 221 surveys sent to healthcare providers, 144 responses were received. Among these, 137 questionnaires were > 80% complete, thus providing a 62% response rate. Most of the responding healthcare providers were female (78%). See Table 1. The respondents included physicians (32%), nurses (32%), and advanced practice providers (17%). Over half of responses (57%) were from individuals who worked at the main campus of the CCC, while the remaining 43% were from those working at community locations. The largest proportion of respondents (44%) had < 5 years of service at our cancer center; 25% had 6–10 years, 12% had 11–15 years, and 9% had 16–20 years, and 10% had > 20 years of service. Respondents treated a variety of cancer types including breast (31%), gastrointestinal (29%), hematologic (23%), lung (21%), and genitourinary (20%). There was approximate 126 responses from those who refer to outpatient PC and 69 responses from those who refer to inpatient PC. Seven out of 10 respondents referred to outpatient PC at least monthly or more, while only about half (48%) referred to inpatient PC at least monthly. About one-sixth (15%) never referred to inpatient PC, compared to 4% who never referred to outpatient PC.

**Table 1:** Demographics

GENDER	N (%)
Female	106 (78%)
Male	29 (21%)
**ROLE**	
Physician	44 (32%)
Nurse	44 (32%)
Advanced Practice	23 (17%)
Social Work	9 (7%)
Other	17 (12%)
**CANCER TYPE**	
Breast	42 (31%)
Gastrointestinal	40 (29%)
General Oncology^a^	40 (29%)
Hematological	31 (23%)
Lung	29 (21%)
Genitourinary	27 (20%)
Melanoma & Sarcoma	24 (18%)
Head & Neck	23 (17%)
Gynecologic	19 (14%)
Stem Cell Transplant	14 (10%)
Sickle Cell	10 (7%)
Neuro Oncology	7 (5%)
Other	8 (6%)
**LOCATION**	
Main Campus	77 (56%)
Community	59 (44%)
**YEARS OF SERVICE**	
< 5	60 (44%)
6–10	34 (25%)
11–15	17 (12%)
16–20	13 (9%)
> 20	13 (9%)

^a^Oncologists who see multiple cancer types as compared to those that only focus on a single cancer types (ex. gastrointestinal cancers)

### Role of palliative care

**Fig. 1:**
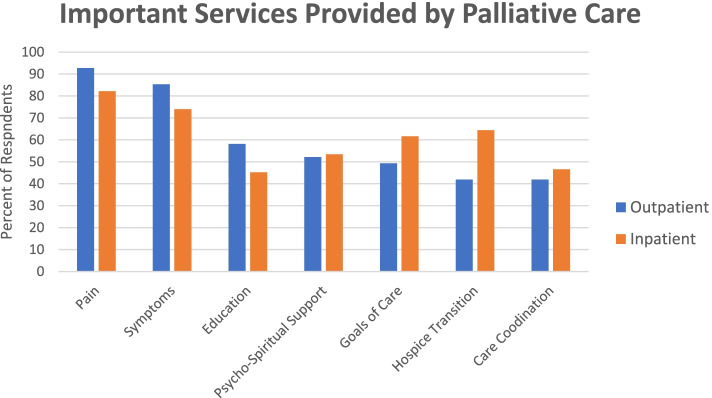
Important services provided by palliative care for outpatient and inpatient settings

Most respondents (82%) agreed that more patients could benefit from palliative/supportive care. There was also a strong consensus that PC is beneficial for outpatients with complex pain (96%) and to a lesser degree, those with complex symptoms other than pain (84%), advanced cancer (80%), and those near the end of life (i.e., less than six-month prognosis; 77%). Similarly, respondents believe that PC is beneficial for inpatients with complex pain (88%), complex symptoms (74%), end-of-life status (71%), and advanced cancer (64%). A similar proportion supported PC for management of patients with unclear goals of care in both inpatient and outpatient settings (50% vs. 46%, respectively). There was a preference among respondents for outpatient over inpatient PC consultation for patients receiving active cancer treatment (65 vs. 51%, *p* = 0.009). In regards to important services provided by PC for outpatients and inpatients, a similar trend was seen in regards to complex pain (93 vs. 82%) and complex symptoms (85% vs. 74%). Transition to hospice and goals of care discussions were valued more for inpatients than for outpatients—64 vs 42% (*p* = 0.007) and 62 vs. 49% (*p* = 0.011), respectively. Emotional and spiritual support and PC education was considered generally important by approximately half of respondents for both inpatient and outpatient settings See Fig. 1.

### Perceived barriers, recommended improvements, and structure

For both outpatient and inpatient referral, lack of availability was identified as the primary barrier (57% and 40%, respectively). Uncertainty about when to refer was cited more commonly for outpatient referrals (19%) than for inpatient referrals (10%). Poor communication (11 vs. 22%), poor alignment with the oncology team’s care plan (16 vs 10%), and lack of trust with PC providers (12 vs 2%) were barriers cited more commonly for inpatient than for outpatient referral.

When asked how PC could better meet their needs, approximately half of respondents indicated a desire for increased availability of inpatient and outpatient PC providers. The next most common responses for both inpatient and outpatient were better continuity between inpatient and outpatient PC and better communication. Addressing advance care planning was emphasized for outpatients (19%) relative to inpatients (13%). Improved alignment between the primary cancer team and palliative care team was emphasized for inpatients (20%) relative to outpatients (12%).

**Fig. 2:**
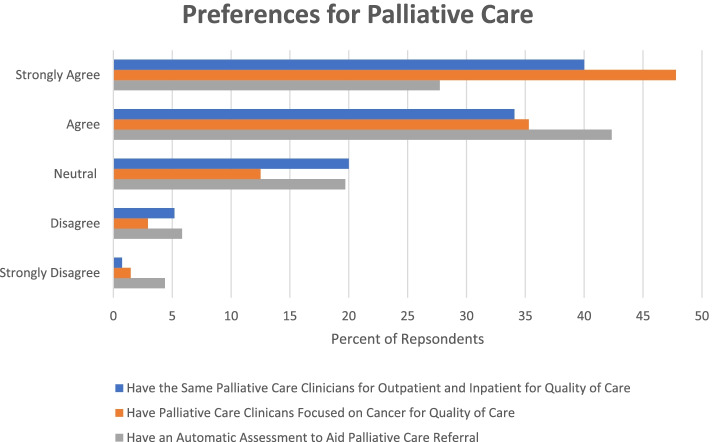
Preferences regarding palliative care team structure and automated assessment tool

Most participants agreed that having the same PC clinicians seeing their patients (both outpatient and inpatient) is important for continuity of care (78%) and quality of care (74%). A large proportion (83%) also agreed that having a team of PC clinicians that focuses on cancer (as compared to a PC team that sees all types of medical conditions) is important for providing high-quality care. Similarly, 79% would prefer to work with a team of PC clinicians that focuses on cancer. A majority of respondents (70%) of respondents felt an automatic assessment tool to help facilitate referrals would be helpful See Fig. 2.

### Multiple regression analysis

In the multiple regression analysis, we found that those who practice at the main campus were significantly more likely than those at community locations to refer to PC ≥ 1x/month (OR 4.2, *p* = 0.003). Those at the main campus were more likely to prefer a cancer focused PC (OR 2.7, *p* = 0.047) as well as those who were general oncologist as compared to those that specialized in a cancer type (OR 2.3, *p* = 0.026). Lastly, those who have practiced at the CCC for ≤ 10 years were more likely to prefer an automatic assessment tool to help aid referrals to PC (OR3.3, *p* = 0.005).

## Discussion

This is the only study in the U.S. that surveyed a variety of oncology providers at a single CCC about attitudes and barriers regarding PC utilization in both inpatient and outpatient settings. The majority of respondents were interested in increasing PC services for their patients. We found differences between the role of outpatient and inpatient PC. Respondents reported that complex pain, complex symptoms, and advanced cancer patients were important services provided by PC in the outpatient setting, and in contrast respondents valued inpatient PC management for goals of care discussions and transition to hospice. The reasons for this significant difference between outpatient and inpatient needs has not been previously identified. The reason for this may be related to the acuity of issues found in the different settings, and that perhaps oncologists are more comfortable and/or able to address goals of care and hospice transition in the outpatient clinic. Additionally, we found that 3 in 4 providers preferred an oncology-focused PC team and felt having the same PC clinicians for outpatient and inpatient was important for continuity of care. The only other similar survey of a variety of oncology healthcare providers gathered opinions about the benefit of early specialist PC at a tertiary cancer center in India [11]. This study reported that oncologists and oncology nurses agreed that early integration of PC in cancer care improves symptom control, end-of-life care, and healthcare-related communication. Compared to nurses, oncologists indicated a greater appreciation for PC interventions in end-of-life care management but not for symptom control or communication.

Surveys of physicians have revealed largely positive attitudes about PC and belief that PC is under-utilized [12–14]. An integrative review of nurses’ attitudes revealed positive views of PC but also a lack of knowledge about PC [15]. One survey of nurses at a U.S. CCC found that oncology nurses had, on average, a strongly positive attitude toward caring for dying patients, though nurses with less experience tended to be less supportive of PC [16]. Our study provides additional evidence that both oncologists and nurses believe that PC interventions improve end-of-life care and symptom management. Contrary to the study by Salins et al., which found a stronger preference for end-of-life PC intervention among oncologists relative to nurses, our study did not find significant differences between opinions of different types of healthcare providers.

The 2017 practice guidelines established by the ASCO include the recommendation that PC for patients with advanced cancer should be delivered through interdisciplinary care teams with consultation available in both outpatient and inpatient settings [5]. While there is evidence supporting the efficacy of such integrated models, there is no published research examining the precise roles of the inpatient and outpatient components of these models and their utilization by oncology health care providers. To date, surveys of healthcare providers have not compared the two settings. Our study revealed some divergence in opinions about the roles of outpatient versus inpatient PC, barriers to those services, and satisfaction. It should be noted that at the time of the survey, the outpatient PC was a cancer focused team while the inpatient PC team was not, which may account for some of the differences found.

Several previous studies have surveyed oncology providers about reasons for PC referral [11, 13, 17–22]. These reasons, which include pain and symptom management, end-of-life care, depression/anxiety, and exhaustion of curative treatment options, suggest desirable roles for PC in the context of oncology. These studies show that PC is valued for pain and symptom relief in both inpatient and outpatient settings. Symptom management was identified as the most important role for PC at the cancer center, which is consistent with results from previous surveys [14, 20, 22]. Of note, only half of respondents reported psych-spiritual support as an important role for the PC service. This could be related to clinicians believing other services were also available to address this need, such as social work or psychiatry, and/or a lack of understanding of the holistic nature of PC. Assisting with goals of care and transition to hospice were noted to be significantly more important for inpatient PC than for outpatient PC. Respondents specified that they prefer to utilize PC to continue care when no other therapy options remain, yet many also agree that early referral is beneficial for patients. Previous surveys have shown that oncology providers largely agree that cancer patients should receive PC early in the disease course, but actual referral practices often fall short of this goal [14, 23]. This discrepancy may be due to the limited availability of PC services and decisions to reserve PC resources for patients at advanced stages of disease who may be most in need.

Systematic reviews have assessed the barriers to specialist PC access, barriers to integration of oncology and PC and factors influencing referrals to specialist PC [8, 9, 24]. The most-reported barriers are system-related and include limited availability of PC services, poor communication between teams, lack of interdisciplinary communication, and low insurance reimbursement. Limited PC availability has been cited as a major barrier to PC referral in several physician surveys, and also found in this study [12, 13, 18, 25]. Provider-related barriers include gaps in knowledge about PC referral practices, fear of estranging patients and families or of deflating hope, belief in incompatibility with ongoing antitumor treatment, lack of time to address PC needs, and stigma associated with the discipline of PC.

Uncertainty about how and when to refer was provided as an additional barrier to PC referral. Most respondents did agree that an automatic assessment and referral built into the electronic medical record would be helpful. Interestingly, it is concordant with the consensus of a panel of international experts that the model of physician referral augmented by automatic referral is an optimal PC referral structure [26]. This is inconsistent with results from other physician surveys, which have shown that oncologists prefer to actively coordinate care and determine the time of PC referral [17]. Respondents showed a strong preference for a PC team with an oncology focus rather than a team that sees other patients in addition to oncology patients. Additionally, most respondents also felt that care would be improved by having the same PC team see patients in both inpatient and outpatient settings.

This study has limitations. Selection bias is possible, though this risk is tempered by the response rate of 62%, which is high for voluntary electronic surveys among healthcare providers. Data from the current study may reflect the opinions of those healthcare providers with an interest in PC. Of note, a significant proportion of respondents had been working at the CCC for < 5 years. Responses to the survey were limited to a single CCC in the Midwestern United States and therefore cannot be generalized out of that setting. The survey tool itself was not validated and this survey was done prior to the COVID pandemic and the increased utilization of telehealth modalities. Lastly, we did not include aspects of home palliative care, which is an important part of the array of services provide for patients [27].

Additional surveys are needed at CCCs to confirm these findings. Evaluations of general PC versus cancer-focused PC are needed to understand the impact of each approach. Future studies are needed to assess patient and family opinions about the role and accessibility of inpatient and outpatient PC, particularly among underserved or disadvantaged patient populations. Additionally, whereas this study gathered opinions of only oncology providers, future studies are needed to understand the perspectives of PC providers. We also need more data about innovative models of integrated PC that improve integration and availability.

## Conclusions

This survey showed that the majority of oncology health care providers believe that more patients could benefit from PC at a CCC. Symptom management was identified as the most important role for PC while assisting with goals of care and transition to hospice were noted to be significantly more important for inpatient PC. Most respondents agree that care would be improved by having the same PC team in both inpatient and outpatient settings. Moreover, respondents showed a strong preference for a cancer-focused PC team. Barriers and areas for improvement include PC availability, improved communication, and alignment and trust with the oncology teams.

## Supplementary Information

**Additional file 1.**

## Data Availability

The datasets generated and/or analyzed during the current study are not publicly available due to institutional guidelines but are available from the corresponding author on reasonable request.
