# Supplementary material for: The perspectives of oncology healthcare providers on the role of palliative care in a comprehensive cancer center
Source: BMC Palliat Care. 2022 Aug 24;21:148. doi: 10.1186/s12904-022-01039-7 (PMC9400235; doi:10.1186/s12904-022-01039-7)
Supplement: Supplementary file 1 — Additional file 1. [file 12904_2022_1039_MOESM1_ESM.pdf]

# **The Perspectives of Oncology Healthcare Providers on the Role of Palliative Care in a Comprehensive Cancer Center**

Survey Supplement

1. Please tell us what type of healthcare provider you are?
    a. Nurse
    b. Advanced Practice Nurse (NP or NCS)
    c. Physician
    d. Social Worker
    e. Other (please specify): ______________

2. What is your cancer team affiliation?
    a. General oncology
    b. Breast
    c. Gastrointestinal (colorectal, pancreas, stomach, esophagus, liver)
    d. Genitourinary (bladder, prostate, kidney)
    e. Gynecological Oncology (ovarian, endometria, cervical)
    f. Head and Neck
    g. Hematological (leukemia, lymphoma, MM)
    h. Lung
    i. Melanoma and Sarcoma
    j. Neuro Oncology
    k. Stem Cell Transplant
    l. Sickle Cell
    m. Other ______________________

3. What location are you primarily affiliated with?
    a. Main Campus
    b. Chagrin
    c. Landerbrook
    d. Westlake
    e. St. John
    f. Sharon
    g. Portage
    h. Other _____________________

4. What is your gender

a. Male
b. Female
c. Other

5. How long have you worked in Seidman Cancer Center?
    a. Less than 5 years
    b. 6 - 10 years
    c. 11 - 15 years
    d. 16 - 20 years
    e. 21 - 25 years
    f. 26 - 30 years
    g. 31 - 35 years
    h. 36 - 40 years
    i. 41+ years

<u>This section asks about your experience with **outpatient** palliative/supportive care</u>

6. How frequently have you referred **outpatients** for palliative/supportive care over the past 3 months?
    a. Never (0 times)
    b. Infrequently (less than once a month)
    c. Moderately Frequently (1-2 times a month)
    d. Frequently (once a week)
    e. Very Frequently (several times a week)
    f. N/A

7. During the past 3 months, how would you rate the <u>coordination of care</u> by the **outpatient** palliative care team?
    a. Poor
    b. Fair
    c. Good
    d. Very Good
    e. Excellent
    f. N/A

8. During the past 3 months, how would you rate the <u>quality of communication</u> from the **outpatient** palliative care team?
    a. Poor
    b. Fair
    c. Good
    d. Very Good

    e. N/A

9. During the past 3 months, how would you rate the quality of care provided by the **outpatient** palliative care team?
    a. Poor
    b. Fair
    c. Good
    d. Very Good
    e. Excellent
    f. N/A

10. What type of your **outpatients** would benefit the most from palliative/supportive care consultation? (check all that apply)
    a. Patients near the end of life (prognosis <6 months)
    b. Patients under active cancer treatment
    c. Patients with advanced cancer (ex. stage IV cancer)
    d. Patients with complex pain
    e. Patients with complex symptoms (other than pain)
    f. Patients that have unclear goals of care
    g. N/A
    h. Other ______________

11. What are the most important services provided by palliative/supportive care for **outpatients**? (check all that apply)
    a. Goals of care discussions
    b. Transition to hospice
    c. Complex pain management
    d. Complex symptom management (other than pain)
    e. Providing emotional and spiritual support
    f. Care coordination
    g. Providing palliative care education
    h. N/A
    i. Other__________

12. What ways could the **outpatient** palliative care team better meet your needs? (check all that apply)
    a. Increase availability
    b. Better communication with the oncology team
    c. Improve alignment with the care plan set by the oncology team
    d. Improve our understanding of cancer care
    e. Better coordination of care
    f. Address advance care planning more
    g. Address goals of care more
    h. Better expertise in pain management
    i. Better expertise in symptom management (other than pain)
    j. Better continuity between inpatient and outpatient palliative care
    k. Provide more emotional and spiritual support
    l. Have a more focused cancer team
    m. N/A
    n. Other _______________

13. What are barriers to referring more patients to the **outpatient** palliative care team? (check all that apply)
    a. Lack of availability
    b. Poor communication
    c. Poor alignment with the oncology team's care plan
    d. Lack of understanding of cancer care
    e. Poor coordination of care
    f. Poor ability to address advance care planning
    g. Poor ability to address goals of care
    h. Poor ability to manage complex pain
    i. Poor ability to manage complex symptoms (other than pain)
    j. Poor continuity of care between inpatient and outpatient palliative care
    k. Poor ability to provide emotional and spiritual support
    l. Lack of trust with palliative care providers
    m. Unsure of when to refer
    n. Unsure how to refer
    o. Lack of time in clinic to make referral
    p. N/A
    q. Other ________________

14. Please rate your overall satisfaction with the **outpatient** palliative care team during the past 3 months.

   a. Not satisfied
   b. Somewhat satisfied
   c. Moderately satisfied
   d. Satisfied
   e. Very satisfied
   f. N/A

<u>This section asks about your experience with **inpatient** palliative/supportive care</u>

15. How frequently have you referred **inpatients** for palliative/supportive care over the past 3 months?
    a. Never (0 times)
    b. Infrequently (less than once a month)
    c. Moderately Frequently (1-2 times a month)
    d. Frequently (once a week)
    e. Very Frequently (several times a week)
    f. N/A

16. During the past 3 months, how would you rate the <u>coordination of care</u> by the **inpatient** palliative care team?
    a. Poor
    b. Fair
    c. Good
    d. Very Good
    e. Excellent
    f. N/A

17. During the past 3 months, how would you rate the <u>quality of communication</u> from the **inpatient** palliative care team?
    a. Poor
    b. Fair
    c. Good
    d. Very Good
    e. Excellent
    f. N/A

18. During the past 3 months, how would you rate the <u>quality of care</u> provided by the **inpatient** palliative care team?
    a. Poor
    b. Fair
    c. Good
    d. Very Good
    e. Excellent
    f. N/A

19. What type of your **inpatients** would benefit the most from palliative/supportive care consultation? (check all that apply)
    a. Patients near the end of life (prognosis <6 months)
    b. Patients under active cancer treatment
    c. Patients with advanced cancer (ex. stage IV cancer)
    d. Patients with complex pain
    e. Patients with complex symptoms (other than pain)
    f. Patients that have unclear goals of care
    g. N/A
    h. Other ______________

20. What are the most important services provided by palliative/supportive care for **inpatients**? (check all that apply)
    a. Goals of care discussions
    b. Transition to hospice
    c. Complex pain management
    d. Complex symptom management (other than pain)
    e. Providing emotional and spiritual support
    f. Providing palliative care education
    g. Care coordination
    h. N/A
    i. Other__________

21. What ways could the **inpatient** palliative care team better meet your needs? (check all that apply)
    a. Increase availability
    b. Better communication with the oncology team
    c. Improve alignment with the care plan set by the oncology team
    d. Improve our understanding of cancer care
    e. Better coordination of care
    f. Address advance care planning more
    g. Address goals of care more
    h. Better expertise in pain management
    i. Better expertise in symptom management (other than pain)
    j. Better continuity between inpatient and outpatient palliative care
    k. Provide more emotional and spiritual support
    l. Have a more focused cancer team
    m. N/A
    n. Other _______________

22. What are barriers to referring more patients to the **inpatient** palliative care team. (check all that apply)
    a. Lack of availability
    b. Poor communication
    c. Poor alignment with the oncology team's care plan
    d. Lack of understanding of cancer care
    e. Poor coordination of care
    f. Poor ability to address advance care planning
    g. Poor ability to address goals of care
    h. Poor ability to manage complex pain
    i. Poor ability to manage complex symptoms (other than pain)
    j. Poor continuity of care between inpatient and outpatient palliative care
    k. Poor ability to provide emotional and spiritual support
    l. Lack of trust with palliative care providers
    m. Unsure of when to refer
    n. Unsure how to refer
    o. Lack of time to make referral
    p. N/A
    q. Other ________________

23. Please rate your overall satisfaction with the **inpatient** palliative care team during the past 3 months.
    a. Not satisfied
    b. Somewhat satisfied
    c. Moderately satisfied
    d. Satisfied
    e. Very satisfied
    f. N/A

<u>Please choose the answer that most represents your opinion about each statement</u>

24. I feel that more of my patients could benefit from palliative/supportive care?
    a. Strongly disagree
    b. Disagree
    c. Neutral
    d. Agree
    e. Strongly Agree

25. Having the same palliative care clinicians seeing my patients both outpatient and inpatient is important for <u>continuity of care</u>?
    a. Strongly disagree
    b. Disagree
    c. Neutral
    d. Agree
    e. Strongly Agree

26. Having the same palliative care clinicians seeing my patients both outpatient and inpatient is important for <u>quality of care</u>?
    a. Strongly disagree
    b. Disagree
    c. Neutral
    d. Agree
    e. Strongly Agree

27. Having a team of palliative care clinicians that <u>focuses on cancer</u> (as compared to a palliative care team that sees all types of medical conditions) is important for my patients to provide <u>high quality care</u>.
    a. Strongly disagree
    b. Disagree
    c. Neutral
    d. Agree
    e. Strongly Agree

28. I would prefer to have a team of palliative care clinicians that <u>focuses on cancer</u> (as compared to a palliative care team that sees all types of medical conditions) taking care of my patients.
    a. Strongly disagree
    b. Disagree
    c. Neutral
    d. Agree
    e. Strongly Agree

29. An automatic assessment tool built into the EMR would be helpful for identifying and facilitating referrals to palliative/supportive care?
    a. Strongly disagree
    b. Disagree
    c. Neutral
    d. Agree
    e. Strongly Agree

<u>Lastly, we would like your feedback about palliative and supportive care</u>

All comments will be confidential and data collected will be anonymous

30. Please share any comments about the positive impact palliative/supportive care has had on your patients.

31. Please share any comments about any challenges you have encountered regarding palliative/supportive care.

32. Please share any comments that might assist with patient care with the implementation of added supportive care capacity in 2019.
